# Signs or Symptoms of Acute HIV Infection in a Cohort Undergoing Community-Based Screening

**DOI:** 10.3201/eid2203.151607

**Published:** 2016-03

**Authors:** Martin Hoenigl, Nella Green, Martha Camacho, Sara Gianella, Sanjay R. Mehta, Davey M. Smith, Susan J. Little

**Affiliations:** Medical University of Graz, Graz, Austria (M. Hoenigl);; University of California San Diego, San Diego, California, USA (M. Hoenigl, N. Green, M. Camacho, S. Gianella, S.R. Mehta, D.M. Smith, S.J. Little);; Veterans Affairs Healthcare System, San Diego (S.R. Mehta, D.M. Smith)

**Keywords:** acute HIV infection, HIV/AIDS and other retroviruses, men who have sex with men, MSM, seroconversion, NAT, screening, acute retroviral syndrome, typical acute retroviral syndrome, signs, symptoms, nucleic acid amplification, testing, viruses

## Abstract

We analyzed signs and symptoms in 90 patients diagnosed with acute HIV infection in a community-based program that offered universal HIV-1 nucleic acid amplification testing. Forty-seven (52%) patients reported ongoing signs or symptoms at the time of testing. Another 25 (28%) reported signs or symptoms that had occurred during the 14 days before testing.

The detection of acute HIV infection (AHI) is critical to HIV prevention and treatment strategies (*1*). Clinical diagnosis of AHI is difficult, however, because the signs and symptoms that occur during seroconversion are frequently not recognized as an indicator of AHI (*2*–*4*). Although screening programs that rely on point-of-care HIV antibody testing will reliably identify persons with established infection, these tests fail to detect AHI (*1*,*5*). The Centers for Disease Control and Prevention began addressing this problem by updating recommendations for the laboratory diagnosis of HIV in healthcare settings to include initial fourth generation HIV-1 p24 antigen–based immunoassays (*6*). However, previous studies indicate that sensitivity of p24 antigen detection for AHI might not exceed 80% (*7*). In addition, most testing programs in nonhealthcare settings continue to rely on routine antibody testing alone, with specific testing for AHI conducted only for persons with signs or symptoms.

Although previous studies focused on retrospective evaluation of AHI symptoms in persons diagnosed with early seropositive HIV infection (*8*,*9*) or cases identified by symptom-based AHI screening, the actual proportion of persons with AHI who are symptomatic at the time of testing remains unknown. We investigated the proportion of persons with AHI who have ongoing or recent signs or symptoms at the time of their diagnostic test in a cohort undergoing community-based universal AHI screening.

## The Study

We analyzed AHI signs and symptoms in 90 patients given a diagnosis of AHI during 2007–2014. As part of this confidential HIV testing program, routine, individual donation, HIV nucleic acid amplification testing (NAT) has been provided to all rapid antibody–negative participants since June 2007 (samples for NAT are obtained at the time of rapid antibody testing) (*7*,*10*,*11*). AHI was defined as having a negative or indeterminate HIV antibody test result in the presence of detectable HIV-1 RNA, corresponding to Fiebig stages I–II, with a mean estimated date of infection within the previous 10 days (95% CI 7–14 days) (*12*). Dates of infection were estimated for all recently infected patients using previously published criteria on the basis of serologic and virologic test results (*13*).

At each patient’s first visit after documentation of AHI diagnosis (median 4 days, interquartile range [IQR] 3–6 days after AHI testing), we obtained blood samples for CD4 and viral load testing and collected detailed information regarding occurrence, duration, and start and stop dates for 11 signs and symptoms associated with AHI (*5*,*14*). Participants were also asked to specify any other symptoms. In addition, patients who participated during 2007–2011 were asked if they had sought medical attention for any signs or symptoms. Typical AHI (i.e., >2 signs/symptoms) was defined according to criteria described by Braun et al. (*14*).

For statistical analysis, SPSS version 21 (SPSS, Inc., Chicago, IL, USA) was used. For analysis on signs or symptoms compatible with AHI, signs or symptoms that started >5 days before the estimated date of infection (i.e., before the 7–14 day 95% CI) were excluded. The University of California San Diego’s Human Research Protections Program approved the study protocol, consent process, and all study-related procedures.

All 90 participants were male and self-identified as men who have sex with men (MSM). Median age was 29 (range 18–67) years. Half (50%) of participants reported white race; 29% reported Hispanic ethnicity. Median number of male partners reported for the previous 12 months was 20 (IQR 14–31). A total of 72 (80%) patients had signs or symptoms associated with AHI that occurred within 2 weeks before undergoing NAT; of these 72 patients, 47 (52% of the study population) had ongoing signs or symptoms, while signs or symptoms had resolved by the time of testing for 25 (28% of the study population). Twelve (13%) reported signs or symptoms starting after testing, while 6 (7%) reported the absence of signs or symptoms (Table 1). A total of 66 patients (73% of the study population) reported headache, pharyngitis, or myalgia occurring during the 14 days before AHI testing.

**Table 1:** Comparison of AHI stage, characteristics of signs or symptoms, CD4+ cell count, and viral load between persons with signs or symptoms before and at the time of NAT versus persons without, San Diego, California, USA, 2007–2014*

Characteristic	Total no. persons	Symptoms before NAT†	Asymptomatic before NAT	p value	Ongoing symptoms at NAT	Absence of symptoms at NAT	p value
No. persons	90	72	18		47	43	
Overall no. signs/symptoms in those symptomatic, median (IQR)	5 (3–7); n = 84	5 (4–7)	5 (2–6); n = 12	NS	6 (4–8)	5 (3–6); n = 37	NS
Duration of symptoms, d, median (IQR)	9 (5–13); n = 79	10 (6–13); n = 67	4 (3–7); n = 12	<0.01	11 (8–14); n = 43	6 (3–9); n = 36	<0.01
CD4+ cell count, cells/μL, median (IQR)	435 (298–597)	435 (302–586)	448 (257–615)	NS	424 (299–592)	445 (295–610)	NS
Viral load, log_10_ RNA, median (IQR)	5.4 (4.5–6.3)	5.8 (4.8–6.4)	4.5 (3.2–5.0)	<0.01	5.6 (4.8–6.4)	5.0 (3.8–6.1)	0.07


*AHI, acute HIV infection; IQR, interquartile range; NAT, nucleic acid amplification testing; NS, not significant. †Most frequently observed signs or symptoms that occurred during the 14 days before NAT or were ongoing at the time of NAT were fatigue (53 persons, 59% of the study population), fever (51, 57%), myalgia (48, 53%), headache (41, 46%), night sweats (35, 39%), pharyngitis (32, 36%), and gastrointestinal symptoms (29, 32%).

Overall, 69 patients (77%) reported signs or symptoms that met criteria of compatibility with AHI (Table 2). Onset of signs or symptoms compatible with AHI occurred at a median of 5 days (IQR 0–8, range –4 to 15 days) after the estimated date of infection. Neither viral load nor CD4 count correlated with duration or actual number of signs or symptoms.

**Table 2:** Signs or symptoms occurring in 69 persons with AHI, San Diego, California, USA, 2007–2014*

Characteristic	Signs/symptoms			p value	Signs/symptoms		p value
	Compatible with AHI†	Resolved before NAT	Ongoing at NAT		Persons seeking medical attention	Persons not seeking medical attention	
No. persons reporting symptoms	69	16	41		10	22	
Duration of symptoms, d, median (IQR)	8 (5–11)	7 (4–8)	11 (7–13)	<0.01	13 (10–17)	10 (4–11)	0.01
No. (%) persons with typical AHI	64 (93)	15 (94)	36 (86)	NS	10 (100)	18 (82)	NS
Signs or symptoms, no. (%)							
Fever	53 (77)	11 (69)	34 (83)	NS	9 (90)	16 (73)	NS
Myalgia	48 (70)	13 (81)	28 (68)	NS	8 (80)	12 (55)	NS
Fatigue	48 (70)	13(81)	29 (71)	NS	9 (90)	13 (59)	NS
Headache	42 (61)	10 (63)	27 (66)	NS	8 (80)	11 (50)	NS
Night sweats	38 (55)	5 (31)	26 (63)	0.04	7 (70)	9 (41)	NS
Pharyngitis	34 (49)	9 (56)	17 (41)	NS	4 (40)	11 (50)	NS
GI symptoms‡	29 (42)	3 (19)	22 (54)	0.02	5 (50)	7 (32)	NS
Rash	19 (28)	6 (38)	12 (29)	NS	5 (50)	5 (23)	NS
Weight loss§	15 (22)	2 (13)	12 (29)	NS	6 (60)	3 (14)	0.01
Arthralgia	14 (20)	3 (19)	8 (20)	NS	4 (40)	1 (5)	0.02


*AHI, acute HIV infection; GI, gastrointestinal; IQR, interquartile range; NAT, nucleic acid amplification testing; NS, not significant. †Defined as having started <4 days before estimated date of infection or after the estimated date of infection. ‡General GI symptoms (e.g., nausea, vomiting, and diarrhea). §Weight loss >2.5 kg.

Data on whether a patient sought medical attention because of signs or symptoms were available for 42 (47%) of 90 patients; of these, 12 (29%) reported that they sought medical attention because of their signs or symptoms and 30 (71%) did not. Significantly higher viral loads were observed for those who sought medical attention compared with those who did not (median 6.1 [IQR 5.7–6.7] log copies/mL vs. 4.7 [IQR 3.4–5.5] log copies/mL; p<0.01).

Overall, 70 (78%) of the 90 patients fulfilled criteria for having typical AHI and 20 (22%) did not (of the latter, 14 had only 1 sign or symptom, and 6 were asymptomatic). Patients with typical AHI had significantly higher viral loads compared with patients without (p<0.01). A total of 61 (85%) of 72 patients with signs or symptoms before NAT testing fulfilled criteria for having typical AHI. In addition, 40 (85%) of 47 patients who had ongoing signs or symptoms at the time of AHI testing fulfilled criteria for having typical AHI at that time.

## Conclusions

We characterized signs or symptoms relative to the date of AHI diagnosis among persons seeking HIV testing in a program offering universal AHI screening. Two findings are notable: 1) 52% of participants reported ongoing signs or symptoms at the time of AHI testing, and 2) 80% reported signs or symptoms occurring within 2 weeks before undergoing testing.

These findings may have major clinical implications for community-based settings that restrict AHI testing to persons with ongoing signs or symptoms. This practice may be relatively insensitive in settings where MSM undergo HIV screening frequently (*11*). Our results show that expansion of AHI screening to include those with signs or symptoms during the 2 weeks before the test may increase the yield of AHI diagnoses by more than half.

Although our results may allow for estimation of sensitivity of signs and symptoms for AHI in persons seeking HIV testing, specificity of signs and symptoms remains unknown (in this study, signs and symptoms were not assessed in those who tested negative, and no control group was available). Estimates on frequency of signs and symptoms in HIV-negative persons (i.e., specificity) ranged widely in previous studies. Although in one study a specificity of 65% was estimated for influenza illness–like symptoms (*15*), specificities ranging from 38% to 91% for recent symptoms were estimated in another study (*5*). Limitations of those studies include the fact that exact time frames for occurrence of signs or symptoms (e.g., ongoing at the time of testing or occurring within the last 14 days) have not been evaluated, which makes comparison of results difficult. A limitation in our study is that all cases of AHI occurred among MSM. Therefore, our results may not be applicable to populations other than MSM, although previous studies have reported that clinical features of AHI may not differ by sex and age of patients (*4*).

In summary, HIV diagnostic testing strategies that limit AHI testing to patients with ongoing signs or symptoms may fail to identify many persons with AHI. In contrast, HIV NAT provided for MSM who report signs or symptoms during the preceding 2 weeks (representing 80% of AHI diagnoses) may increase the yield of AHI diagnoses by more than half.
